# Establishment of an Organogenesis-Based Regeneration System and Induction of Somatic Embryogenesis in *Catalpa ovata*

**DOI:** 10.3390/plants15081177

**Published:** 2026-04-10

**Authors:** Pingan Bao, Xingping Huo, Jingshuang Sun, Guanzheng Qu, Wenjun Ma, Junhui Wang, Ruiyang Hu

**Affiliations:** 1 State Key Laboratory of Tree Genetics and Breeding, Experimental Center of Forestry in North China, Chinese Academy of Forestry, Beijing 102300, China; pinganb888@163.com (P.B.);; 2 State Key Laboratory of Tree Genetics and Breeding, Northeast Forestry University, Harbin 150040, China; 3 College of Horticulture Science & Technology, Hebei Normal University of Science & Technology, Qinhuangdao 066004, China; 4 State Key Laboratory of Tree Genetics and Breeding, Research Institute of Forestry, Chinese Academy of Forestry, Beijing 100091, China

**Keywords:** *Catalpa ovata*, zygotic embryo, regeneration system, somatic embryogenesis, Trichostatin A (TSA), genotype specificity

## Abstract

To overcome the seasonal constraints of explant availability and facilitate genetic improvement in *Catalpa ovata*, this study established a dual-pathway in vitro regeneration system (encompassing adventitious shoot organogenesis and somatic embryogenesis) using mature zygotic embryos. We systematically evaluated the synergistic effects of maternal genotypes, plant growth regulators (PGRs), basal media, and the histone deacetylase inhibitor Trichostatin A (TSA). Genotype screening revealed significant divergence in regenerative potential, with the half-sib family 32F17 exhibiting superior responsiveness (84.7% callus induction). A high cytokinin-to-auxin ratio (ZA3 medium) optimally drove direct shoot organogenesis. For adventitious shoot proliferation, the addition of TDZ significantly improved the multiplication coefficient (up to 2.99 on ZB4 medium), although a physiological trade-off with shoot elongation was observed. In parallel, the application of 10 µM TSA significantly enhanced somatic embryogenesis from embryogenic calli, effectively alleviating the inhibitory constraints of exogenous PGRs. For rhizogenesis, the DKW basal medium proved superior to half-strength MS, with the ZE3 treatment (0.1 mg·L^−1^ NAA + 0.1 mg·L^−1^ IBA) yielding the highest rooting frequency (69.6%) and robust root architecture. Notably, while somatic embryo conversion remained recalcitrant, plantlets derived exclusively from the adventitious shoot organogenesis pathway were successfully acclimatized ex vitro. These transplanted plantlets exhibited consistently high survival rates (83.1–84.4%) across all tested genotypes, effectively overcoming the initial genotype-dependent recalcitrance. Collectively, this optimized protocol provides a reliable technical platform for the large-scale clonal propagation and biotechnological breeding of *C. ovata*.

## 1. Introduction

*Catalpa ovata* G. Don is a deciduous tree species endemic to China and is widely used in urban landscaping and ecological restoration due to its elegant crown architecture, conspicuous inflorescences, and strong adaptability to infertile soils and adverse environmental conditions [1]. In addition to its ornamental and ecological value, *C. ovata* tissues are rich in bioactive compounds, including acacetin glycosides and flavonoids, which have demonstrated anti-inflammatory, detoxifying, and skin-related biological activities, highlighting its medicinal and functional potential [2,3,4]. However, the large-scale clonal propagation and biotechnological breeding of *C. ovata* are severely constrained by inefficient conventional vegetative methods, such as cutting and grafting, which are typically limited by strong seasonal dependence, poor rooting ability, and low multiplication efficiency [5].

Plant tissue culture has been successfully applied to numerous woody species as an efficient alternative propagation strategy [6,7,8]. Compared with sexual propagation, in vitro regeneration avoids problems associated with low fertility, progeny segregation, and unstable germination, while offering higher multiplication rates and shorter production cycles than traditional vegetative propagation methods. More importantly, regeneration through somatic embryogenesis or adventitious organogenesis enables the production of genetically uniform plantlets, providing essential technical support for large-scale clonal propagation, rapid expansion of elite genotypes, and genetic transformation and functional genomics research in woody plants [9,10,11].

The establishment of an efficient regeneration system is influenced by multiple interacting factors, among which explant source, genotype background, and the type and concentration of exogenous plant growth regulators (PGRs) are considered critical determinants [12]. Auxins such as 2,4-dichlorophenoxyacetic acid (2,4-D), α-naphthaleneacetic acid (NAA), and indole-3-butyric acid (IBA) are commonly used to induce callus formation and embryogenic competence, whereas cytokinins including 6-benzylaminopurine (6-BA), zeatin (ZT), and thidiazuron (TDZ) are primarily involved in maintaining embryogenic potential and promoting shoot regeneration [13,14,15,16]. The ratio between auxins and cytokinins plays a pivotal role in directing developmental pathways, with high auxin-to-cytokinin ratios favoring embryogenic callus induction and high cytokinin-to-auxin ratios promoting adventitious shoot formation through activation of shoot meristem–related genes such as *WUSCHEL* [17,18]. During callus induction, substantial interspecific differences exist in both the choice of basal media and the composition and concentration of PGRs. For instance, in *Paeonia suffruticosa* ‘Hongxialanman’, a callus induction rate of 94.4% was achieved from petiole explants cultured on Murashige and Skoog (MS) medium supplemented with 1.0 mg·L^−1^ TDZ and 0.1 mg·L^−1^ NAA [19]. In *Paeonia ostii* ‘Fengdan’, an embryogenic callus induction rate as high as 97.3% was obtained on WPM medium containing 0.1 mg·L^−1^ 6-BA and 2.5 mg·L^−1^ 2,4-D [20]. In *Punica granatum* L., callus induction on MS medium supplemented with 1.0 mg·L^−1^ 6-BA and 1.0 mg·L^−1^ NAA reached approximately 72% [21]. Collectively, these findings highlight the pronounced species-specificity of callus induction systems, underscoring the necessity of optimizing basal media and PGR combinations for different plant materials.

Within the genus *Catalpa*, in vitro regeneration has been explored, though significant bottlenecks remain, particularly for *C. ovata*. To date, in vitro propagation of *C. ovata* has been largely restricted to direct organogenesis, exhibiting a relatively low regeneration frequency (maximum 30%) on Schenk and Hildebrandt (SH) or Woody Plant (WP) media, which was strongly dependent on the application of cytokinins like ZT and 6-BA alongside the auxin indole-3-acetic acid (IAA) [22]. In contrast, more advanced somatic embryogenesis systems have been established in the related species *C. bungei* by employing specific basal media and PGR combinations. For example, a callus induction rate of 45.7% was reported on Driver and Kuniyuki Walnut (DKW) medium supplemented with 2.0 mg·L^−1^ 6-BA, 1.0 mg·L^−1^ ZT, 0.1 mg·L^−1^ NAA, 30 g·L^−1^ sucrose, and 3 g·L^−1^ Phytagel [7]. Additionally, high-frequency callus induction (90%) from immature zygotic embryos was achieved using half-strength MS medium supplemented with 1.0 mg·L^−1^ 2,4-D and 0.1 mg·L^−1^ 6-BA, followed by SE induction using a modified ratio of 6-BA and NAA [23]. Furthermore, the successful induction of embryogenic calli across multiple genotypes using mature seeds and stem segments has confirmed that regeneration efficiency in *Catalpa* is heavily influenced by the specific explant source and the precise formulation of MS or DKW basal media with appropriate PGRs [24]. Building upon the successful basal media (MS and DKW) and PGR profiles (auxins like 2,4-D and NAA; cytokinins like 6-BA and ZT) identified in these prior *Catalpa* studies, we systematically optimized their concentrations and combinations in the present study. By doing so, we aimed to overcome the regeneration recalcitrance of *C. ovata*, significantly improve the embryogenic callus induction rate, and establish a foundational, high-efficiency regeneration protocol.

In addition to hormonal regulation, epigenetic modifications have emerged as critical regulators of cellular totipotency. Histone deacetylation, mediated by histone deacetylases (HDACs), generally restricts cell fate reprogramming by compacting chromatin structure, whereas inhibiting HDAC activity can enhance regenerative competence [25]. Trichostatin A (TSA), a potent HDAC inhibitor, has been reported to promote somatic embryogenesis in various plant species [26]. Mechanistically, TSA treatment induces histone hyperacetylation, which leads to a relaxed chromatin state. This structural change facilitates the transcriptional activation of essential embryogenic pathways that are otherwise silenced during somatic development [27,28,29]. Although this epigenetic regulation has been well-documented in model systems like *Arabidopsis thaliana* and barley [30,31], its application in woody plants—particularly in ornamental tree species such as *C. ovata*—remains limited, and the potential of TSA to overcome regeneration recalcitrance in these species is poorly understood.

Therefore, in this study, we established a high-efficiency regeneration system for *C. ovata* using mature zygotic embryos from five half-sib families. Beyond optimizing genotype and PGR combinations, we introduced the histone deacetylase inhibitor TSA to investigate its potential in enhancing embryogenic competence. This work aims to integrate somatic embryogenesis and organogenesis pathways, providing a robust technical platform for the genetic improvement and mass propagation of this valuable woody species.

## 2. Results

### 2.1. Effects of Genotypes and PGRs on Callus Induction and Morphogenesis

**Figure 1:**
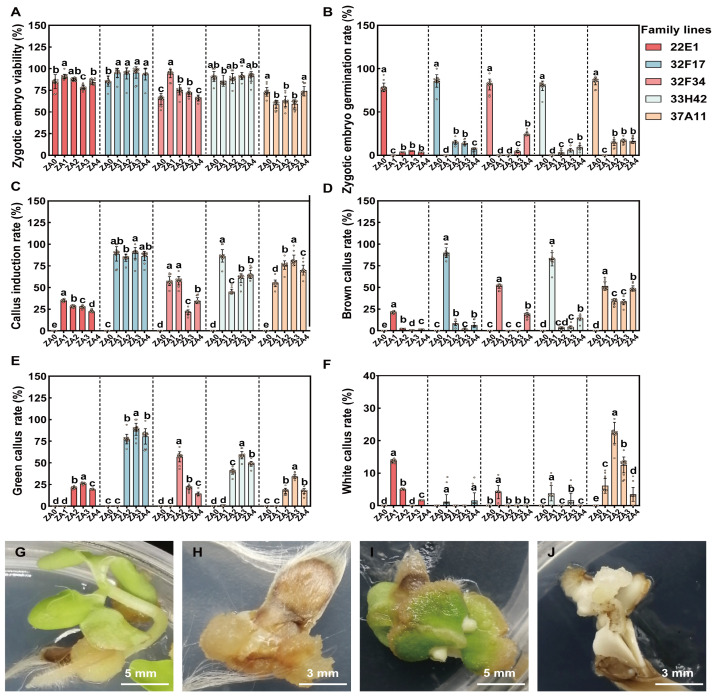
Effects of PGRs and family genotypes on the dedifferentiation and morphological fate of *C. ovata* zygotic embryos. (**A**–**F**) Statistical analysis of physiological responses among five half-sib families (22E1, 32F17, 32F34, 33H42, and 37A11) across different induction media. ZA0 serves as the PGR-free control medium. ZA1–ZA4 represent treatments with varying PGR combinations. (**A**) Zygotic embryo viability; (**B**) Zygotic embryo germination rate; (**C**) Callus induction rate; (**D**) Brown callus rate; (**E**) Green callus rate; (**F**) White callus rate. Bars represent the mean ± standard deviation (SD) of 15 biological replicates (*n* = 15), and open circles indicate individual biological replicates. Different lowercase letters denote statistically significant differences among treatments within each family line (*p* < 0.05), as determined by factorial ANOVA followed by Duncan’s multiple range test. (**G**–**J**) Representative appearances of the explants. (**G**) Direct germination of the zygotic embryo into a seedling (observed across all treatments, but occurring at an exceptionally high frequency in the PGR-free ZA0 medium); (**H**) Severe browning and necrosis of callus (commonly observed in the ZA1 treatment); (**I**) High-quality green compact callus with high organogenic potential (predominantly in the ZA3 treatment); (**J**) White friable callus.

To elucidate the synergistic regulatory effects of PGRs and genotypes on the dedifferentiation and developmental fates of *C. ovata* zygotic embryos, this study systematically evaluated the differential responses of five half-sib families across various induction media (ZA0–ZA4) (Figure 1). The results indicated that zygotic embryos from all tested families maintained high basal viability (overall viability > 75%, Figure 1A), precluding explant quality as a confounding factor in induction efficiency. On the control medium lacking exogenous PGRs (ZA0), the zygotic embryos germinated (e.g., family 32F17 exhibited a germination rate of approximately 85%, Figure 1B,G). Conversely, the introduction of exogenous PGRs (ZA1–ZA4) served as a critical trigger for developmental reprogramming, significantly suppressing direct germination and successfully initiating callus formation (Figure 1C). Notably, callus induction and subsequent callus appearance exhibited profound genotype dependency and PGR specificity. Family 32F17 demonstrated exceptional regenerative competence, achieving a total callus induction rate under PGR treatments that was significantly higher than those of the other families (*p* < 0.001). Regarding the developmental fate of the callus, the precise PGR ratio played a decisive role. Although the ZA1 treatment successfully initiated dedifferentiation, it provoked severe tissue necrosis, with the browning rates of families 32F17 and 33H42 surging to 80–90% (Figure 1D,H). Following the optimization of the cytokinin proportion, the ZA3 treatment emerged as the optimal formulation for inducing the target appearance: green compact callus. On this medium, the proportion of green callus possessing high organogenic potential reached its maximum (Figure 1E,I); specifically, the induction rate for family 32F17 approached 90%, and tissue browning was effectively mitigated (Figure 1D). In contrast, other suboptimal treatments occasionally produced a certain proportion of non-embryogenic white callus (Figure 1F,J). However, as PGR concentrations were further elevated in the ZA4 treatment, the green callus rate exhibited a declining trend, suggesting the onset of physiological inhibition by supra-optimal PGR levels. Collectively, these findings substantiate that family 32F17 represents the elite donor germplasm for scalable in vitro regeneration, and that the ZA3 medium establishes the optimal PGR milieu for the organogenesis of high-quality green embryogenic callus.

### 2.2. Effects of Initial PGR Treatments and Subculture Medium on Morphogenesis

To investigate the morphogenic potential of calli under different PGR backgrounds, the calli formed during the initial induction phase (ZA1–ZA4) were subcultured onto ZA3 (containing 0.2 mg·L^−1^ NAA) and ZA5 (containing 0.2 mg·L^−1^ 2,4-D) media for comparative analysis. The results indicated that on the ZA3 medium containing NAA, the calli successfully initiated organogenesis, though the efficiency was highly dependent on the initial induction background (Table 1). Calli subjected to the initial ZA4 induction exhibited the strongest regenerative capacity upon transfer to ZA3 (ZA4→ZA3). Specifically, within family 32F17, the adventitious shoot induction rate peaked at 55.0 ± 4.1% under this treatment combination, accompanied by the highest rooting rate of 40.0 ± 4.9%. Furthermore, the early ZA1 induction environment conferred a specific developmental fate on the calli; only calli initially formed on ZA1 were capable of differentiating into somatic embryos after transfer to ZA3 (e.g., reaching 19.1 ± 3.3% and 20.0 ± 2.8% in families 32F17 and 37A11, respectively).

In stark contrast to the organogenic responses observed on ZA3, the ZA5 subculture medium containing 2,4-D exhibited a strong inhibitory effect on organ differentiation (Table 2). The induction rates for both adventitious shoots and roots were 0% across all tested families on this medium, indicating that 2,4-D effectively maintained the cells in a dedifferentiated state. However, the potential for somatic embryogenesis was not affected by this inhibition. Calli initially formed on the ZA1 medium stably expressed the capacity for somatic embryo differentiation even after being transferred to ZA5 (with induction rates of 16.1 ± 1.8% and 16.7 ± 2.5% for families 32F17 and 37A11, respectively).

Taken together, these subculture results demonstrate that the type of auxin in the subculture medium (NAA vs. 2,4-D) dictates whether the organogenesis program can be successfully initiated, whereas the initial PGR induction environment predetermines the specific developmental potential of the cells. Specifically, the early ZA1 induction environment is a prerequisite for initiating somatic embryogenesis, while an initial high-concentration cytokinin treatment (ZA4) combined with NAA during the subculture phase (ZA3) represents the optimal strategy for adventitious shoot regeneration.

### 2.3. Effects of Family Genotypes and PGRs on Shoot Proliferation and Elongation

**Figure 2:**
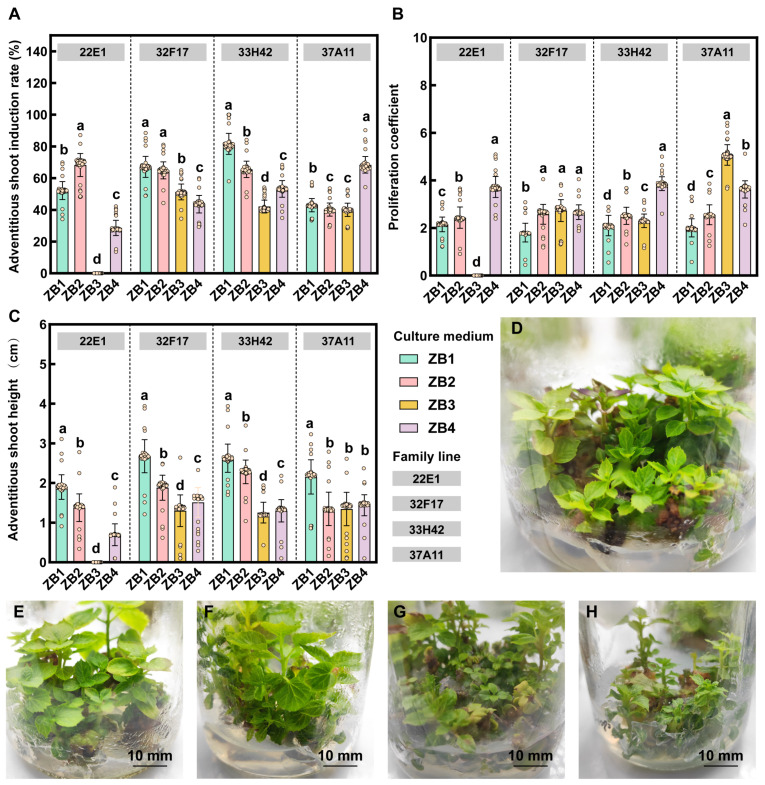
Effects of proliferation media (ZB1–ZB4) and family genotypes on the multiplication and elongation of adventitious shoots in *C. ovata*. (**A**–**C**) Statistical analysis of physiological responses among four half-sib families (22E1, 32F17, 33H42, and 37A11) across different proliferation media (ZB1–ZB4). (**A**) Adventitious shoot induction rate; (**B**) Proliferation coefficient; (**C**) Adventitious shoot height. Bars represent the mean ± standard deviation (SD) of 15 biological replicates (*n* = 15), and open circles denote individual biological replicates. Different lowercase letters denote statistically significant differences among treatments within each family line (*p* < 0.05), as determined by factorial ANOVA followed by Duncan’s multiple range test. (**D**–**H**) Representative morphological observations of adventitious shoots. (**D**) Overall morphological view of robust adventitious shoots; (**E**) Shoot morphology on ZB1 medium; (**F**) Shoot morphology on ZB2 medium; (**G**) Shoot morphology on ZB3 medium; (**H**) Shoot morphology on ZB4 medium. The comparisons across E–H illustrate the physiological trade-off between proliferation and elongation (i.e., elongated and robust on ZB1/ZB2 vs. highly clustered but stunted on ZB3/ZB4).

To optimize the multiplication efficiency of adventitious shoots and evaluate the genotypic effect, regenerated adventitious shoots were subcultured onto different proliferation media (ZB1–ZB4). The results indicated that the proliferation and elongation of adventitious shoots exhibited significant differences among families and were strongly regulated by the ratio of exogenous PGRs (Figure 2). Regarding the adventitious shoot induction rate, the families displayed specific responses to the PGR treatments: families 33H42 and 32F17 exhibited higher induction rates on the ZB1 medium, whereas family 37A11 reached its induction peak on the ZB4 medium (Figure 2A). More importantly, a significant physiological trade-off was observed between the multiplication quantity and elongation quality of the adventitious shoots. With the adjustment of specific PGR combinations (ZB3 and ZB4), the proliferation coefficient of adventitious shoots was significantly enhanced (e.g., family 37A11 achieved the highest proliferation coefficient on ZB3, Figure 2B); however, this process was accompanied by a severe inhibition of shoot elongation (Figure 2C). Morphological observations clearly corroborated these data trends: on ZB3 and ZB4 media, the adventitious shoots exhibited a highly dense, stunted, and clustered morphology (Figure 2G,H). Conversely, although the ZB1 medium yielded a moderate proliferation coefficient in certain families, it greatly promoted shoot elongation; all tested families achieved their maximum adventitious shoot heights on ZB1 (Figure 2C), producing robust regenerated plantlets with properly expanded leaves (Figure 2D,E). These results suggest that in the large-scale in vitro propagation of *C. ovata*, specific proliferation PGR combinations must be tailored to different families, and a two-step culture strategy of “proliferation first (e.g., ZB3/ZB4) followed by elongation (ZB1)” may be required in practical applications.

### 2.4. Epigenetic Modulation of Somatic Embryogenesis by TSA

**Figure 3:**
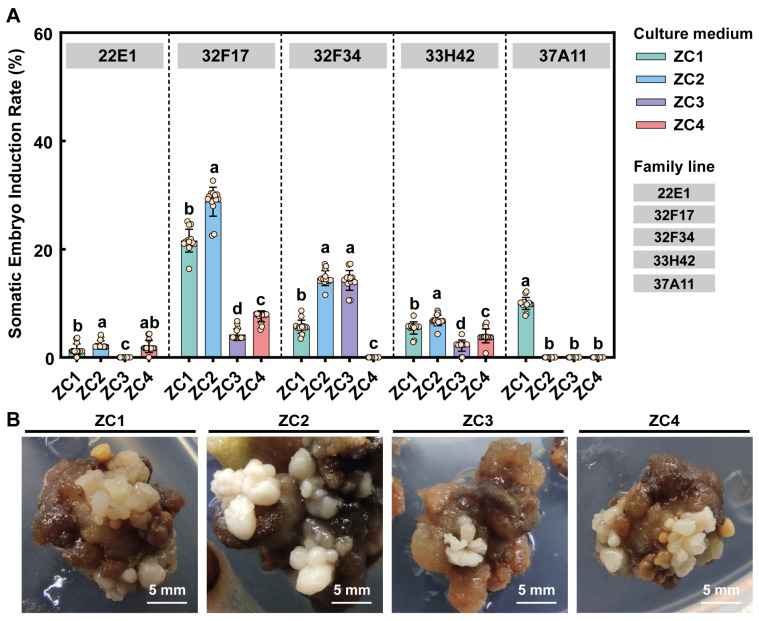
Synergistic effects of TSA and PGRs on somatic embryogenesis across different *C. ovata* families. (**A**) Statistical analysis of somatic embryo induction rates among five half-sib families under different culture media (ZC1: PGR-free and TSA-free; ZC2: 10 μM TSA only; ZC3: PGRs only; ZC4: PGRs + 10 μM TSA). Bars represent the mean ± standard deviation (SD) of 15 biological replicates (*n* = 15), and open circles denote individual biological replicates. Different lowercase letters denote statistically significant differences among treatments within each family line (*p* < 0.05), as determined by factorial ANOVA followed by Duncan’s multiple range test. (**B**) Representative morphological observations of somatic embryogenesis in family 32F17 across the four treatments, highlighting the abundant formation of high-quality somatic embryos under the ZC2 treatment.

While exogenous PGRs successfully directed adventitious shoot organogenesis, the induction of somatic embryogenesis remained highly recalcitrant and strictly dependent on the specific early induction environment. To overcome this limitation and further unlock the embryogenic potential of the calli, the HDAC inhibitor TSA was introduced to evaluate its epigenetic regulatory effects (ZC1–ZC4) (Figure 3). The results demonstrated that TSA significantly enhanced the frequency of somatic embryogenesis, particularly in a PGR-free microenvironment. As shown in Figure 3A, when calli were cultured on the PGR-free control medium (ZC1), a spontaneous SE induction rate of approximately 21.6% was observed in the highly responsive family 32F17. However, the introduction of exogenous PGRs (ZC3) severely inhibited this process, causing the induction rate for 32F17 to plummet to below 4.2%. Strikingly, the addition of 10 μM TSA alone (ZC2) exhibited a potent inductive capacity, maximizing the SE induction rate for 32F17 to nearly 28.8%, which was significantly higher than that of the ZC1 control (*p* < 0.001). Similar promoting effects of ZC2 were also observed in families 32F34 and 33H42. Furthermore, even under the suppressive background of exogenous PGRs (ZC4), the supplementation of TSA partially alleviated the developmental inhibition, yielding induction rates significantly higher than those of the ZC3 treatment across multiple families (Figure 3A). Morphological observations (Figure 3B) confirmed that high-quality, distinct globular and cotyledonary somatic embryos were most abundantly formed under the ZC2 treatment. These findings robustly demonstrate that epigenetic modulation via TSA can effectively override the inhibitory constraints of exogenous PGRs, thereby serving as a powerful catalyst for somatic embryogenesis in *C. ovata*.

### 2.5. Effects of Family Genotypes, PGRs, and Basal Media on Rhizogenesis

**Figure 4:**
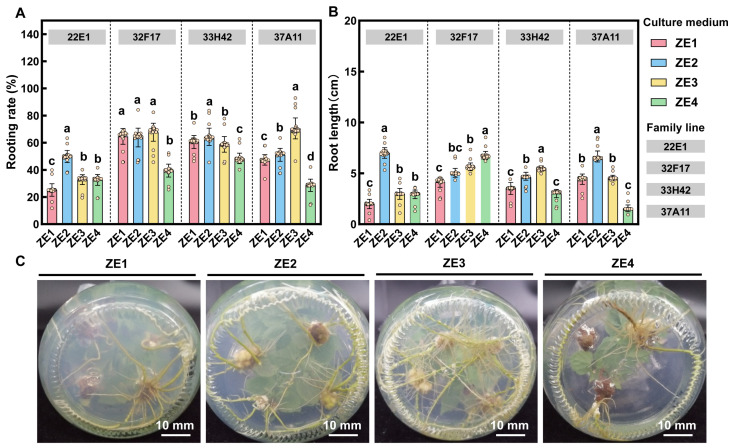
Effects of basal media, PGR combinations, and family genotypes on the adventitious rooting of *C. ovata*. (**A**,**B**) Statistical analysis of rooting rate (**A**) and root length (**B**) among four half-sib families (22E1, 32F17, 33H42, and 37A11) under different treatments (ZE1: DKW + 0.2 mg·L^−1^ NAA; ZE2: DKW + 0.2 mg·L^−1^ IBA; ZE3: DKW + 0.1 mg·L^−1^ NAA + 0.1 mg·L^−1^ IBA; ZE4: 1/2 MS + 0.1 mg·L^−1^ NAA + 0.1 mg·L^−1^ IBA). Bars represent the mean ± standard deviation (SD) of 15 biological replicates (*n* = 15), and open circles denote individual biological replicates. Different lowercase letters denote statistically significant differences among treatments within each family line (*p* < 0.05), as determined by factorial ANOVA followed by Duncan’s multiple range test. (**C**) Representative morphological observations of root system architecture from the bottom view across the four treatments, demonstrating the robust root development on the ZE3 medium compared to the stunted growth on the ZE4 medium.

To optimize the rhizogenesis of regenerated adventitious shoots, the effects of family genotypes, PGR combinations, and basal media (DKW vs. 1/2 MS) were systematically evaluated (ZE1–ZE4) (Figure 4). The rooting capacity exhibited significant genotypic variation; for instance, family 32F17 demonstrated the highest overall rooting rates across DKW-based media (ZE1–ZE3), whereas family 22E1 showed relatively lower responsiveness (Figure 4A). Beyond genotypic differences, the synergistic application of exogenous PGRs and the choice of basal medium profoundly impacted the root system architecture. While single-auxin treatments (ZE1 with 0.2 mg·L^−1^ NAA or ZE2 with 0.2 mg·L^−1^ IBA) successfully induced roots, the combination of both auxins in the ZE3 treatment (DKW + 0.1 mg·L^−1^ NAA + 0.1 mg·L^−1^ IBA) yielded the most optimal rooting performance for elite families like 33H42 and 37A11, promoting both high rooting rates and robust root elongation (Figure 4A,B). Crucially, when comparing basal media under identical PGR supplementation, the DKW medium (ZE3) proved significantly superior to the conventional 1/2 MS medium (ZE4). For most families, the ZE4 treatment led to a sharp decline in rooting rates and severely restricted root elongation (Figure 4A,B). Morphological observations further corroborated these findings: root systems developed on the ZE3 medium were thick, well-branched, and robust, whereas those on ZE4 appeared sparse and underdeveloped (Figure 4C). These results underscore that the specific mineral nutrient composition of DKW, acting synergistically with a balanced auxin combination, is essential for the formation of a high-quality root system in *C. ovata*, thereby ensuring high survival rates during subsequent ex vitro acclimatization.

### 2.6. Ex Vitro Acclimatization

**Figure 5:**
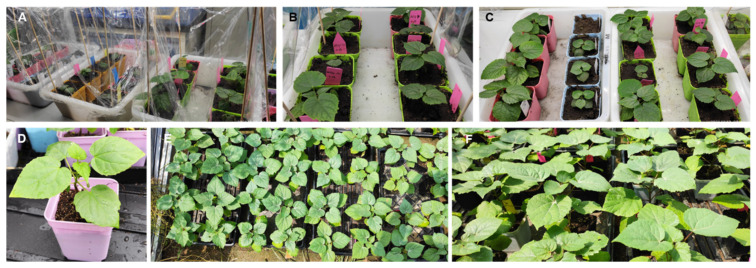
Acclimatization and subsequent ex vitro growth performance of *C. ovata* tissue-cultured plantlets. (**A**) Initial acclimatization stage under plastic film with high humidity; (**B**,**C**) Adaptive and robust growth in the greenhouse after film removal; (**D**) Detailed view of an individual plantlet showing a lignified stem and normal leaf morphology; (**E**,**F**) Growth performance of the plantlet population in the nursery at the late stage of acclimatization. Notably, all acclimatized plantlets shown here were derived exclusively from the adventitious shoot organogenesis pathway.

Mature zygotic embryos ultimately developed into morphologically normal whole plants exclusively via the adventitious shoot organogenesis pathway. Notably, although high-quality somatic embryos were successfully induced under TSA mediation, their germination and plantlet conversion remained highly recalcitrant under the current in vitro conditions, frequently arresting at late developmental stages. Consequently, all regenerated plantlets selected for ex vitro acclimatization and transplantation in this study were strictly derived from the adventitious shoot organogenesis route (Figure 5).

Robust regenerated plantlets with well-developed root systems formed on the optimal rooting medium were selected for greenhouse transplantation. The transplanted plantlets exhibited robust adaptability and healthy growth, characterized by progressive stem lignification, proportionate leaf expansion, and significant increases in plant height. Long-term observations confirmed no significant macroscopic morphological differences between the in vitro regenerated plants and conventional seed-derived seedlings, and no obvious somaclonal variation was observed. More crucially, as shown in Table 3, the regenerated plantlets from the five half-sib families all demonstrated exceptionally stable and high survival rates during the ex vitro acclimatization phase, ranging from 83.1% to 84.4%. In stark contrast to the high genotype dependency observed during the initial in vitro induction phase, the acclimatization survival rates exhibited no significant variance among the different families. These quantitative data robustly demonstrate that the optimized rooting and hardening protocols established in this study can effectively overcome the regeneration discrepancies caused by diverse maternal genetic backgrounds, ensuring high survival proportions across different genotypes. In conclusion, this organogenesis-based regeneration system possesses high stability and broad applicability, providing a solid technical foundation for the large-scale clonal propagation of elite *C. ovata* germplasm.

## 3. Discussion

### 3.1. Strategic Selection of Zygotic Embryos and Genotypic Dependence

In this study, an efficient dual regeneration system integrating embryogenic and organogenic pathways was established for *C. ovata*. The selection of mature zygotic embryos (ZEs) as primary explants was a strategic choice to ensure a stable, year-round resource, overcoming the seasonal constraints typically associated with immature embryos in *Catalpa* species [7,23]. Furthermore, our results reaffirm that genotype is a primary determinant of recalcitrance in woody plant regeneration [32,33,34]. Although ZEs from cross-pollinated *C. ovata* represent a diverse genotypic pool rather than clones, this diversity facilitated the identification of elite lines (e.g., family 32F17) with exceptional regenerative competence. Such family-specific variations are likely attributable to differences in endogenous phytohormone homeostasis and genetic backgrounds, providing a crucial technical foundation for future mass propagation of elite *Catalpa* germplasm.

### 3.2. Impact of PGR Balance on Callus Quality and Browning Control

The dedifferentiation trajectory of *C. ovata* is strictly governed by the exogenous PGR balance. We observed that high auxin-to-cytokinin ratios maximize induction but are frequently accompanied by severe tissue browning. This phenomenon is primarily attributed to the potent activity of synthetic auxins (e.g., 2,4-D), which can provoke stress responses in woody explants, leading to phenolic oxidation and subsequent necrosis [17,20,35]. By optimizing the PGR profile with specific cytokinin combinations, such as ZT (0.2 mg·L^−1^) and 6-BA (0.6 mg·L^−1^), we effectively mitigated this browning while improving callus viability and morphogenic quality. This transition from focusing on “induction frequency” to “callus health” is a critical advancement over earlier *C. ovata* protocols, providing a robust foundation for subsequent regeneration pathways [22].

### 3.3. PGR Legacy and Epigenetic Reprogramming of Somatic Embryogenesis

Embryogenic competence in *C. ovata* appears to be imprinted during the initial induction phase―a phenomenon termed “PGR legacy.” The requirement for early exposure to 2,4-D to establish embryogenic potential suggests that this auxin triggers specific regulatory networks (e.g., *LEC* genes) that irreversibly redirect cell fate [36,37,38]. While 2,4-D sustains this trajectory, other auxins like NAA favor organogenesis [36,39,40,41]. A pivotal breakthrough in our study is the use of TSA to override the inhibitory constraints of exogenous PGRs. As a histone deacetylase inhibitor, TSA likely promotes cell reprogramming by increasing histone acetylation at key embryogenesis-related loci, thereby releasing transcriptional repression [42]. This epigenetic modulation proved more effective than traditional PGR adjustments alone, aligning with conserved mechanisms reported in *Arabidopsis* and conifers [30,43]. This evidence demonstrates that targeting epigenetic barriers can effectively compensate for the physiological age of mature explants, facilitating high-frequency SE induction. However, it is important to note that the current discussion regarding TSA-induced histone hyperacetylation is primarily inferred from conserved mechanisms in model species such as *Arabidopsis*. Future molecular and epigenetic investigations are required to definitively confirm these specific regulatory mechanisms in *C. ovata*.

### 3.4. Physiological Trade-Offs in Shoot Organogenesis and Elongation

During adventitious shoot proliferation, we identified a striking trade-off between multiplication and quality. The use of highly active phenylurea derivatives (e.g., TDZ) aggressively drives shoot proliferation but simultaneously suppresses internode elongation, likely due to the antagonism of gibberellin-mediated pathways [44]. Our proposed two-step strategy—prioritizing multiplication followed by an elongation phase using milder cytokinins (e.g., ZT)—offers a practical solution to the common issues of vitrification and stunted growth in *Catalpa* micropropagation [43,45,46]. This approach ensures that regenerated plantlets possess the morphological vigor required for subsequent rooting.

### 3.5. Optimization of Rhizogenesis and Root System Architecture

As a typical hardwood tree species, *C. ovata* exhibits severe recalcitrance to spontaneous in vitro rooting. Therefore, rather than evaluating PGR-free controls, our rooting experiments focused primarily on the synergistic optimization of basal media and exogenous auxins to construct a robust root system. Successful rhizogenesis requires a synergistic balance between mineral nutrition and exogenous auxin signaling. The superiority of the DKW medium over 1/2 MS in our system suggests that the specific mineral composition of DKW is better suited for the high nutrient demands of *C. ovata* root development. Furthermore, the application of dual-auxin combinations promoted a more balanced and robust root system architecture than single-auxin treatments, which often trigger excessive callus formation and poor morphology in woody species [47]. The establishment of this high-quality rooting protocol ensures high survival rates during ex vitro acclimatization, completing a robust technical platform for *Catalpa* genetic improvement.

## 4. Materials and Methods

### 4.1. Plant Material and Explant Preparation

Mature siliques were harvested from five half-sib families of *C*. *ovata* (designated as 22E1, 32F17, 32F34, 33H42, and 37A11) located in the germplasm repository of Mentougou District, Beijing, China (116°06′ E, 39°56′ N). These families were specifically selected based on the superior cold tolerance of their maternal lines. In this study, these five half-sib families represent distinct maternal genetic backgrounds (genotypes), serving as a primary variable to systematically evaluate genotypic effects on in vitro regeneration. Seeds were surface-sterilized by immersion in 75% (*v*/*v*) ethanol for 1 min, followed by treatment with 0.1% (*w*/*v*) HgCl_2_ (supplemented with 1–2 drops of Tween-20) for 10 min under continuous agitation. After being rinsed five times with sterile distilled water, mature zygotic embryos (ZEs) were meticulously excised as primary explants.

### 4.2. General Basal Medium and Culture Conditions

To avoid redundancy in subsequent descriptions, a standardized basal medium and environmental regime were applied across all experimental stages unless otherwise specified. All culture media were formulated using the DKW basal platform, supplemented with 30 g·L^−1^ sucrose and solidified with 7 g·L^−1^ agar [48,49]. The pH of all media was adjusted to 5.8 prior to autoclaving at 121 °C for 20 min.

Unless a dark treatment was specifically required (e.g., initial induction phases), all in vitro cultures were maintained in a controlled growth chamber at 26 ± 1 °C under a 16 h light/8 h dark photoperiod provided by cool-white fluorescent tubes with a photosynthetic photon flux density (PPFD) of 30–50 μmol·m^−2^·s^−1^.

### 4.3. Callus Induction and Morphogenesis

Excised ZEs were inoculated horizontally into 90 mm sterile Petri dishes containing the standardized DKW callus induction media supplemented with various PGR combinations (ZA1–ZA4, Table 4). To promote cellular dedifferentiation, these initial cultures were maintained in total darkness for 30 d. At the end of the induction period, the callus induction frequency was quantified as the percentage of explants exhibiting visible callus formation, and the appearance (e.g., color, vigor, and texture) of the calli was documented.

### 4.4. Callus Subculture and Shoot Proliferation

Following the 30-day initial induction, to evaluate the carry-over effects of primary PGR treatments on subsequent morphogenesis, all primary calli (from ZA1–ZA4) were partitioned and transferred onto two subculture media with distinct physiological orientations (Table 5):

#### 4.4.1. Divergent Subculture Pathways

Following the initial 30 d of culture on the induction media (ZA1–ZA4), the responding explants were subcultured onto ZA3 and ZA5 media for adventitious shoot proliferation. The proliferation performance was evaluated and recorded after 15 d of subculture.

Shoot Induction Group (ZA3): The first set of calli was transferred to ZA3 medium, which features a high cytokinin-to-auxin ratio designed to trigger the organogenic pathway for adventitious shoot formation.

Embryogenic Maintenance Group (ZA5): Simultaneously, the second set was transferred to ZA5 medium containing the potent synthetic auxin 2,4-D. This treatment was specifically intended to suppress premature differentiation, sustain embryogenic competence, and generate high-quality donor material for the somatic embryogenesis experiments described in Section 4.5.

#### 4.4.2. Optimization of Shoot Proliferation

For shoots successfully regenerated via the ZA3 pathway, nodal segments (approximately 1.5–2.0 cm in length) were excised and inoculated onto shoot proliferation media (ZB1–ZB4, Table 6). This experimental phase aimed to systematically evaluate the synergistic effects of various cytokinin concentrations (TDZ and ZT) on the multiplication coefficient (mean number of new shoots per explant) and internode elongation (shoot height) across the four responding half-sib families (22E1, 32F17, 33H42, and 37A11).

### 4.5. TSA-Mediated Somatic Embryogenesis

To explore the epigenetic modulation of somatic embryogenesis (SE), vigorous embryogenic calli derived from the ZA5 lineage were subcultured onto induction media (ZC1–ZC4, Table 7) supplemented with or without 10 μM TSA and specific PGRs. Cultures were initially incubated in darkness to induce proembryogenic masses and were subsequently transferred to the standard photoperiod upon the appearance of globular embryos. After 30 d, the SE induction efficiency was determined as the percentage of callus clumps producing distinct somatic embryos.

### 4.6. Adventitious Root Induction

Robust individual shoots (approximately 3.0 cm in height) generated from the proliferation phase were transferred to rooting media (ZE1–ZE4, Table 8) to identify optimal conditions for rhizogenesis. The experimental design evaluated the efficacy of the standardized DKW versus half-strength MS (1/2 MS) basal media, as well as the synergistic effects of combined auxins (NAA and IBA) [50]. After 30 d, the rooting percentage and mean root length were recorded.

### 4.7. Ex Vitro Acclimatization and Transplantation

Regenerated plantlets possessing a robust root system measuring 2.0–3.0 cm were selected for ex vitro acclimatization in a greenhouse facility. The hardening process commenced by loosening the culture vessel caps for 3–5 d under natural illumination to facilitate gradual adaptation to ambient conditions. Subsequently, the plantlets were carefully extracted, and the root zone was gently rinsed with tepid water to eliminate residual agar before being transplanted into plastic plug trays filled with a sterilized substrate matrix of peat, perlite, and vermiculite (3:1:1). During the initial post-transplant phase, a microenvironment with high relative humidity (RH > 85%) and controlled temperature (26 ± 1 °C) was maintained using transparent plastic covers. Ventilation and light intensity were progressively increased over 2 weeks prior to the complete removal of the protective covers. Thirty days post-transplantation, the survival rate and vegetative growth recovery were evaluated. To ensure the reproducibility of the results, the ex vitro acclimatization and transplantation experiments were conducted in four independent batches at different times. The survival rate for each family was calculated based on the outcomes of these four separate experimental trials (*n* = 4).

### 4.8. Statistical Analysis

All experiments were conducted following a completely randomized design (CRD) with 15 biological replicates per treatment (*n* = 15). Prior to statistical evaluation, all percentage data (e.g., induction frequencies and rooting rates) were subjected to an arcsine square root transformation to ensure data normality and homogeneity of variance. Data were analyzed using factorial analysis of variance (ANOVA) to assess the main effects and interactions of genotypes and treatments. Significant differences were separated using Duncan’s multiple range test (DMRT). All statistical tests were performed at a significance level of *p* < 0.05 using SPSS version 25.0 software (SPSS Inc., Chicago, IL, USA). Data in all figures and tables are presented as the mean ± standard deviation (SD), and significant differences are indicated by different lowercase letters.

## 5. Conclusions

This study successfully established a dual-pathway regeneration system (encompassing both adventitious shoot organogenesis and somatic embryogenesis) for *C. ovata* utilizing mature zygotic embryos, thereby overcoming the seasonal constraints typically associated with explant availability. We demonstrated that maternal genotype is a primary determinant of in vitro recalcitrance, identifying the half-sib family 32F17 as an elite germplasm. The precise modulation of PGRs dictated developmental pathways: a high cytokinin-to-auxin ratio effectively drove direct shoot organogenesis, whereas sustained 2,4-D exposure was crucial for maintaining embryogenic competence. A pivotal breakthrough was the application of the epigenetic modulator TSA (10 µM), which successfully overcame developmental barriers and significantly promoted somatic embryo differentiation from mature tissues. Furthermore, optimizing the balance of cytokinins resolved the physiological trade-off between shoot multiplication and elongation, while the superiority of the DKW basal medium ensured robust rhizogenesis and high ex vitro survival rates. Despite these advancements, further refinement of the regeneration system is warranted. Future research should investigate the embryogenic potential of zygotic embryos across various developmental stages (e.g., immature embryos) and focus on improving the maturation and plantlet conversion efficiency of somatic embryos. Collectively, this robust protocol resolves the regeneration bottleneck in *C. ovata*, laying a vital foundation for future genetic transformation, haploid breeding, and elite germplasm preservation.

## Figures and Tables

**Table 1:** Effects of initial induction background and family genotype on the morphogenic potential of calli subcultured on ZA3 medium.

Treatment	Family Line	Non-Dedifferentiation Rate (%)	Rate of Callus Induction with Different Color (%)				Shooting Rate (%)	Somatic Embryogenesis (%)	Rooting Rate (%)
			White	Light-Green	Light-Yellow	Dark Brown			
ZA1→ZA3	22E1	10.0 ± 2.1 a	13.3 ± 2.7 a	0 c	76.7 ± 5.2 b	0 e	0 a	3.3 ± 1.5 b	0 a
ZA1→ZA3	32F17	0 c	0 c	0 c	50.0 ± 1.9 c	50.0 ± 4.0 b	0 a	19.1 ± 3.3 a	0 a
ZA1→ZA3	32F34	0 c	0 c	0 c	92.9 ± 5.8 a	7.1 ± 3.7 d	0 a	0 c	0 a
ZA1→ZA3	33H42	2.2 ± 2.4 b	2.2 ± 1.9 b	6.7 ± 2.6 b	73.3 ± 3.2 b	15.6 ± 1.7 c	0 a	0 c	0 a
ZA1→ZA3	37A11	0 c	0 c	10.0 ± 1.6 a	20.0 ± 2.3 d	70.0 ± 4.8 a	0 a	20.0 ± 2.8 a	0 a
ZA2→ZA3	22E1	60.4 ± 3.4 a	0 a	16.3 ± 2.4 c	23.3 ± 2.9 a	0 d	6.9 ± 1.7 c	0 a	2.3 ± 1.6 c
ZA2→ZA3	32F17	0 d	0 a	64.3 ± 4.8 ab	0 c	35.7 ± 2.4 b	32.1 ± 3.0 a	0 a	10.7 ± 2.2 b
ZA2→ZA3	32F34	16.7 ± 4.7 b	0 a	66.6 ± 7.9 a	0 c	16.7 ± 3.1 c	0 d	0 a	0 d
ZA2→ZA3	33H42	14.6 ± 4.2 b	0 a	18.8 ± 1.9 c	0 c	66.6 ± 6.1 a	22.9 ± 3.3 b	0 a	16.7 ± 2.3 a
ZA2→ZA3	37A11	6.7 ± 3.2 c	0 a	60.0 ± 3.8 b	20.0 ± 3.3 b	13.3 ± 2.3 c	6.7 ± 3.3 c	0 a	0 d
ZA3→ZA3	22E1	19.1 ± 3.6 a	0 a	31.0 ± 3.5 c	4.8 ± 2.9 b	45.1 ± 3.8 c	2.4 ± 2.5 d	0 a	2.4 ± 2.0 c
ZA3→ZA3	32F17	0 c	0 a	17.7 ± 2.4 d	29.4 ± 2.4 a	52.9 ± 3.8 b	47.1 ± 3.7 a	0 a	23.5 ± 2.6 a
ZA3→ZA3	32F34	0 c	0 a	44.4 ± 4.5 b	0 d	55.6 ± 2.7 b	11.1 ± 3.1 c	0 a	0 d
ZA3→ZA3	33H42	15.4 ± 3.7 b	0 a	15.4 ± 1.7 d	7.7 ± 1.2 c	61.5 ± 3.0 a	21.2 ± 2.0 b	0 a	7.7 ± 2.8 b
ZA3→ZA3	37A11	0 c	0 a	72.7 ± 4.6 a	0 d	27.3 ± 4.0 d	0 e	0 a	0 d
ZA4→ZA3	22E1	7.7 ± 2.1 b	3.9 ± 2.0 b	0 c	11.5 ± 2.1 b	76.9 ± 7.4 b	3.9 ± 2.6 d	0 a	3.9 ± 1.9 c
ZA4→ZA3	32F17	0 c	0 c	45.0 ± 5.6 b	45.0 ± 3.5 a	10.0 ± 3.4 d	55.0 ± 4.1 a	0 a	40.0 ± 4.9 a
ZA4→ZA3	32F34	11.1 ± 1.2 a	11.4 ± 2.3 a	77.5 ± 9.2 a	0 d	0 e	22.2 ± 2.3 c	0 a	11.1 ± 2.9 b
ZA4→ZA3	33H42	8.2 ± 1.6 b	0 c	0 c	8.2 ± 1.6 c	83.6 ± 5.0 a	28.6 ± 2.2 b	0 a	10.2 ± 1.4 b
ZA4→ZA3	37A11	0 c	0 c	44.5 ± 2.9 b	11.1 ± 1.2 b	44.4 ± 2.1 c	0 e	0 a	0 d

Note: “Treatment” indicates the sequential transfer of calli from the induction to the subculture medium. Data are presented as mean ± standard deviation (SD) of 15 biological replicates (*n* = 15). Different lowercase letters within the same column indicate significant differences among family genotypes within the same specific treatment block (*p* < 0.05, Duncan’s multiple range test).

**Table 2:** Effects of initial induction background and family genotype on the developmental fate of calli subcultured on ZA5 medium.

Treatment	Family Line	Non-Dedifferentiation Rate (%)	Rate of Callus Induction with Different Color (%)				Shooting Rate (%)	Somatic Embryogenesis (%)	Rooting Rate (%)
			White	Light-Green	Light-Yellow	Dark Brown			
ZA1→ZA5	22E1	28.9 ± 2.8 a	0 b	2.2 ± 1.0 a	68.9 ± 8.7 b	0 b	0 a	2.2 ± 1.1 c	0 a
ZA1→ZA5	32F17	5.9 ± 3.9 d	0 b	0 b	94.1 ± 3.9 a	0 b	0 a	16.1 ± 1. 8 a	0 a
ZA1→ZA5	32F34	7.3 ± 4.3 d	0 b	0 b	92.7 ± 4.3 a	0 b	0 a	0 d	0 a
ZA1→ZA5	33H42	2.4 ± 1.5 c	2.4 ± 2.2 a	0 b	90.4 ± 3.4 a	4.8 ± 1.5 a	0 a	4.8 ± 0.9 b	0 a
ZA1→ZA5	37A11	5.6 ± 1.1 b	0 b	0 b	88.8 ± 5.0 a	5.6 ± 1.1 a	0 a	16.7 ± 2.5 a	0 a
ZA2→ZA5	22E1	6.1 ± 1.3 d	6.1 ± 1.1 a	6.1 ± 2.2 c	12.2 ± 2.8 c	69.5 ± 3.3 a	0 a	0 b	0 a
ZA2→ZA5	32F17	8.0 ± 1.6 c	0 b	8.0 ± 3.9 c	20.0 ± 1.6 b	64.0 ± 5.3 ab	0 a	0 b	0 a
ZA2→ZA5	32F34	16.7 ± 2.3 a	0 b	33.3 ± 3.6 a	0 d	50.0 ± 6.1 d	0 a	0 b	0 a
ZA2→ZA5	33H42	10.4 ± 2.2 b	0 b	29.2 ± 3.8 b	0 d	60.4 ± 4.8 bc	0 a	2.1 ± 1.9 a	0 a
ZA2→ZA5	37A11	0 e	0 b	0 d	41.7 ± 3.3 a	58.3 ± 6.5 c	0 a	0 b	0 a
ZA3→ZA5	22E1	4.6 ± 1.8 c	0 a	2.3 ± 2.5 b	27.3 ± 2.0 b	65.8 ± 6.3 b	0 a	0 a	0 a
ZA3→ZA5	32F17	0 d	0 a	33.3 ± 3.8 a	9.5 ± 2.1 d	57.2 ± 5.5 c	0 a	0 a	0 a
ZA3→ZA5	32F34	8.3 ± 2.6 b	0 a	0 c	33.3 ± 2.4 a	58.4 ± 7.6 c	0 a	0 a	0 a
ZA3→ZA5	33H42	13.8 ± 3.2 a	0 a	3.5 ± 2.3 b	8.6 ± 2.3 d	74.1 ± 7.1 a	0 a	0 a	0 a
ZA3→ZA5	37A11	0 d	0 a	33.3 ± 3.0 a	22.2 ± 2.7 c	44.5 ± 5.2 d	0 a	0 a	0 a
ZA4→ZA5	22E1	14.9 ± 3.4 a	4.3 ± 2.9 a	14.9 ± 3.4 c	23.4 ± 1.4 c	42.5 ± 3.3 b	0 a	0 a	0 a
ZA4→ZA5	32F17	3.9 ± 2.2 b	3.9 ± 1.2 a	34.5 ± 2.8 a	34.6 ± 1.5 a	23.1 ± 2.4 c	0 a	0 a	0 a
ZA4→ZA5	32F34	0 c	0 c	11.1 ± 3.2 d	11.1 ± 2.2 e	77.8 ± 8.4 a	0 a	0 a	0 a
ZA4→ZA5	33H42	0 c	0 c	6.7 ± 2.4 e	15.0 ± 1.8 d	78.3 ± 6.7 a	0 a	0 a	0 a
ZA4→ZA5	37A11	0 c	2.4 ± 0.8 b	23.8 ± 1.1 b	28.6 ± 3.7 b	45.2 ± 2.6 b	0 a	0 a	0 a

Note: “Treatment” indicates the sequential transfer of calli from the induction to the subculture medium. Data are presented as mean ± standard deviation (SD) of 15 biological replicates (*n* = 15). Different lowercase letters within the same column indicate significant differences among family genotypes within the same specific treatment block (*p* < 0.05, Duncan’s multiple range test).

**Table 3:** Ex vitro acclimatization and survival rates of regenerated *C. ovata* plantlets among different families.

Family Line	Total No. of Plants Subjected to Acclimatization	No. of Surviving Plantlets	Mean Survival Rate (%)
32F17	314	261	83.1% ± 1.3 a
33H42	139	117	84.2% ± 3.4 a
22E1	70	59	84.2% ± 2.9 a
32F34	71	59	83.3% ± 3.2 a
37A11	58	49	84.4% ± 2.7 a

Note: Data are presented as mean ± standard deviation (SD) of four independent transplanting batches (*n* = 4). Total number of plants represents the cumulative sum across all four batches. The same lowercase letter “a” indicates no significant difference among the family lines (*p* > 0.05, Duncan’s multiple range test).

**Table 4:** Composition of PGRs for callus induction.

Treatment	PGRs			
	2,4-D (mg⋅L^−1^)	6-BA (mg⋅L^−1^)	ZT (mg⋅L^−1^)	NAA (mg⋅L^−1^)
ZA0	–	–	–	–
ZA1	0.6	–	0.2	–
ZA2	–	0.6	–	0.2
ZA3	–	0.6	0.2	0.2
ZA4	–	1.2	0.4	0.4

Note: “–“ indicates the absence of the specific growth regulator.

**Table 5:** PGR combinations for divergent subculture pathways (adventitious shoot organogenesis vs. embryogenic maintenance).

Treatment	PGRs			
	2,4-D (mg⋅L^−1^)	6-BA (mg⋅L^−1^)	ZT (mg⋅L^−1^)	NAA (mg⋅L^−1^)
ZA3	–	0.6	0.2	0.2
ZA5	0.2	0.6	0.2	–

Note: “–“ indicates the absence of the specific growth regulator.

**Table 6:** PGR combinations for adventitious shoot proliferation.

Treatment	PGRs			
	6-BA (mg⋅L^−1^)	ZT (mg⋅L^−1^)	TDZ (mg⋅L^−1^)	NAA (mg⋅L^−1^)
ZB1	0.6	0.2	–	0.1
ZB2	0.6	–	0.2	0.1
ZB3	0.6	0.2	0.2	0.1
ZB4	1.2	0.4	0.4	0.1

Note: “–“ indicates the absence of the specific growth regulator.

**Table 7:** Combinations of epigenetic modulator (TSA) and PGRs for somatic embryo induction.

Treatment	PGRs			TSA (μM)
	2,4-D (mg⋅L^−1^)	6-BA (mg⋅L^−1^)	ZT (mg⋅L^−1^)	
ZC1	–	–	–	–
ZC2	–	–	–	10
ZC3	0.1	0.3	0.1	–
ZC4	0.1	0.3	0.1	10

Note: “–“ indicates the absence of the specific growth regulator.

**Table 8:** Basal media and PGR combinations for adventitious root induction.

Treatment	Basal Medium	PGRs	
		NAA (mg·L^−1^)	IBA (mg·L^−1^)
ZE1	DKW	0.2	–
ZE2	DKW	–	0.2
ZE3	DKW	0.1	0.1
ZE4	1/2MS	0.1	0.1

Note: “–“ indicates the absence of the specific growth regulator.

## Data Availability

Data are contained within the article.
